# Caregiver burden in professionals working in different settings for persons with dementia: Zuzana Katreniakova

**DOI:** 10.1093/eurpub/ckac130.116

**Published:** 2022-10-25

**Authors:** Z Katreniakova, M Nemcikova, I Nagyova

**Affiliations:** Department of Social and Behavioural Medicine, PJ Safarik University in Kosice, Faculty of Medicine & SAVEZ, Kosice, Slovakia

## Abstract

**Introduction:**

Professionals caring for persons with dementia (PwD) have to meet demands from various sides. Considering today's high standards put on institutional care with respect to “person centred care” and the skills related to specific needs of PwD, caring for care staff is one of the major challenges.

**Objectives:**

The aim of this study was to investigate the caregiver burden in professional dementia care and its work related differences.

**Methods:**

The study was carried out from September 2021 to February 2022 among 105 professionals working in different settings for PwD in Slovakia including home care and day care centres. The Professional Care Team Burden scale (PCTB) and the Zarit Burden Interview (ZBI-12) were chosen to measure caregiver burden. Length of work in the organisation (< 3, 3 to 6, > 6 years), job position (domiciliary and other care worker), and current dementia care intensity (≤ 8, 9 to 39, > 40 hours/week) were also measured. Independent samples T-tests and ANOVA were used to analyse the differences (IBM SPSS 27).

**Results:**

87.6% of professionals were women (mean age 48.6±9.8 years). 52.5% worked more than 6 years in the organisation, 53.3% were in the job position of domiciliary care workers, and for 52.1% the current dementia care intensity was 9 to 39 hours/week. Caregiver burden mean scores achieved were 26.7±4.0 (PCTB) and 9.7±6.2 (ZBI-12). The significant difference was found in the PCTB by job position with the higher burden in domiciliary care workers (mean score 26.0 + 4.2, p < 0.05). No significant differences were observed in the PCTB and the ZBI-12 by the length of work in the organisation and current dementia care intensity.

**Conclusions:**

Specific scales for assessing professionals’ caregiver burden are useful to uncover areas for intervention. Structuring the interventions by taking the care staff subjective feelings of burden into account is important for future improvements in institutional care. (Grant support: VEGA no. 1/0372/20).

**Key messages:**

- Caring for professionals is one of the major challenges to improve health and social services for persons with dementia.
- Structuring the tailored interventions should take the caregiver burden into account.

